# Empowering families by engaging and relating Murri way: a grounded theory study of the implementation of the Cape York Baby Basket program

**DOI:** 10.1186/s12884-015-0543-y

**Published:** 2015-05-21

**Authors:** Janya McCalman, Andrew Searles, Roxanne Bainbridge, Rachael Ham, Jacki Mein, Johanna Neville, Sandra Campbell, Komla Tsey

**Affiliations:** The Cairns Institute and College of Public Health and Tropical Medicine, James Cook University, PO Box 6811, Cairns, QLD 4870 Australia; Health Research Economist, Hunter Medical Research Institute and University of Newcastle, Locked Bag 1000, New Lambton, 2305, NSW Australia; Apunipima Cape York Health Council, PO Box 12045, Westcourt, 4870 QLD Australia; Centre of Research Excellence in Prevention of Chronic Conditions, James Cook University, PO Box 6811, Cairns, 4870 QLD Australia; The Cairns Institute and College of Arts and Society and Education, James Cook University, PO Box 6811, Cairns, 4870 QLD Australia

**Keywords:** Maternal and child health, Indigenous, Aboriginal and Torres Strait Islander, Intervention, Grounded theory, Antenatal care, Postnatal care, Remote

## Abstract

**Background:**

Evaluating program outcomes without considering how the program was implemented can cause misunderstandings and inefficiencies when initiating program improvements. In conjunction with a program evaluation, reported elsewhere, this paper theorises the process of implementing an Indigenous Australian maternal and child health program. The Baby Basket program was developed in 2009 for the remote Cape York region and aimed to improve the attendance and engagement of Indigenous women at antenatal and postnatal clinics through providing three baskets of maternal and baby goods and associated health education.

**Methods:**

Constructivist grounded theory methods were used to generate and analyse data from qualitative interviews and focus groups with Indigenous women who received the baskets, their extended family members, and healthcare workers who delivered them. Data was coded in NVivo with concepts iteratively compared until higher order constructs and their relationships could be modelled to explain the common purpose for participants, the process involved in achieving that purpose, key strategies, conditions and outcomes. Theoretical terms are italicised.

**Results:**

Program implementation entailed *empowering families* through a process of *engaging and relating Murri* (Queensland Indigenous) *way*. Key influencing conditions of the social environment were *the remoteness of communities*, *keeping up with demand*, *families’ knowledge, skills and roles* and *organisational service approaches and capacities. Engaging and relating Murri way* occurred through four strategies: *connecting through practical support, creating a culturally safe practice, becoming informed and informing others*, and *linking at the clinic*. These strategies resulted in women and families *taking responsibility for health* through *making healthy choices, becoming empowered health consumers* and *advocating for community changes*.

**Conclusions:**

The theoretical model was applied to improve and revise Baby Basket program implementation, including increased recognition of the importance of *empowering families* by extending the home visiting approach up to the child’s third birthday. *Engaging and relating Murri way* was strengthened by formal recognition and training of Indigenous health workers as program leaders. This theoretical model of program implementation was therefore useful for guiding program improvements, and could be applicable to other Indigenous maternal and child health programs.

## Background

Despite global improvements in maternal and child health outcomes, Indigenous people worldwide still experience much poorer maternal and child health outcomes than non-Indigenous populations [1]. In Australia, for example, significant reductions in Aboriginal and Torres Strait Islander (hereafter Indigenous) child mortality occurred over the 40 year period between 1967 and 2006 [2]. However, for the Australian states and territories for which data is available^a^, in 2010, Indigenous child mortality remained more than double that of the non-Indigenous population (with 45 compared with 20 deaths per 100,000 children aged 1–4 years) [3]. In 2008, the Australian Government recognised that improving maternal and child health also had the potential to play an important role in creating the foundations for improved Indigenous health and wellbeing throughout the lifetime [4] and pledged to halve the gap in mortality rates for children under five by 2018 [5].

Further reducing maternal and child health mortality requires government investment in innovative models to improve access to and the quality of Indigenous maternal and child health services and programs. Reviews of Indigenous Australian maternal and child health programs have documented evaluations of intervention models and a diversity of antenatal and postnatal program and service components [6-8]. Successful outcomes of Indigenous maternal and child health programs were attributed to common contributing factors such as the presence of Indigenous and female staff, outreach, home-visiting and transport. As well, reviews cited the value of programs being community-based and implemented by community controlled health organisations [7,8]. However, consistent with a review of Indigenous health promotion tools which found that only 30% publications reported intended or actual implementation processes [9], few of the Indigenous Australian maternal and child health studies reviewed had reported how programs were implemented [8,10].

A clear understanding of program implementation allows the connection of program strategies, observed outcomes and ultimately the measurement of impact. This ‘line of sight’ also provides valuable information to allow others to adopt or adapt successful programs. This paper provides a theoretical framework which explains the process by which the Baby Basket program was implemented across eleven remote communities. The Baby Basket program aimed to engage Murri (Indigenous Australians that traditionally occupied most of modern-day Queensland) women from Cape York with the health system through encouraging early and frequent attendance at antenatal clinics and regular postnatal check-ups. Through enhanced engagement, the hypothesised impact was better maternal health; reduced complications during and after pregnancy; an increased proportion of normal weight babies; and thriving infants. Complementing a quantitative outcome evaluation of the Baby Basket program [11], this paper responds to the research question: How was the Baby Basket program implemented in routine primary health care practice?

## Methods

### Design of the study

A phronetic strengths-based approach was used with a concern for values and interests beyond analytical, scientific and technical rationality and a solutions-based rather than problem-saturated focus [12,13]. Constructivist grounded theory was chosen as the most suitable method due to its ability to explain program implementation and its appropriateness for Indigenous Australian settings [12,14,15]. In particular, in the context of Indigenous Australian research, the concurrent data collection and analysis requisite in grounded theory methods avoided unnecessary data collection from Indigenous people who have been historically over researched [15]. The intent of grounded theory is to build a substantive theory from the practical experience and knowledge of those directly involved in the phenomena under study. The theory comprises a common purpose and process, strategies and consequences, all of which are influenced by the conditions of the social environment [16,17]. Apunipima Cape York Health Council approved the project and ethics approval was granted by the James Cook University Ethics Committee (H5321).

### The setting

Cape York is a remote northern region of Queensland with 51% (8566) Indigenous residents, and poor maternal and child morbidity and mortality [18,19]. The eleven discrete Indigenous communities of Cape York were historically formed as mission settlements, with Indigenous people relocated to them in the late 1800s. Each has a distinctive history, tribal population groups and church influence. Since the 1980s, most communities have been governed by incorporated community councils which have full control over the administration of local government services [20]. Many Indigenous residents experience socioeconomic disadvantage, with low levels of skilled occupations, high rates of unemployment and low incomes. These factors contribute to more than double the number of low birth weight babies in comparison to the rest of Queensland and high infant mortality levels (11 infant deaths per 1,000 live births) [19]. Families are impacted by the common health determinants of harmful alcohol consumption, smoking, overweight and obesity, poor nutrition, physical inactivity, and risk factors for mental health. In 2005/06, 70% of pregnant women in Cape York were reported to have smoked at some time during their pregnancy and there were high rates of gestational diabetes [19].

**Figure 1:**
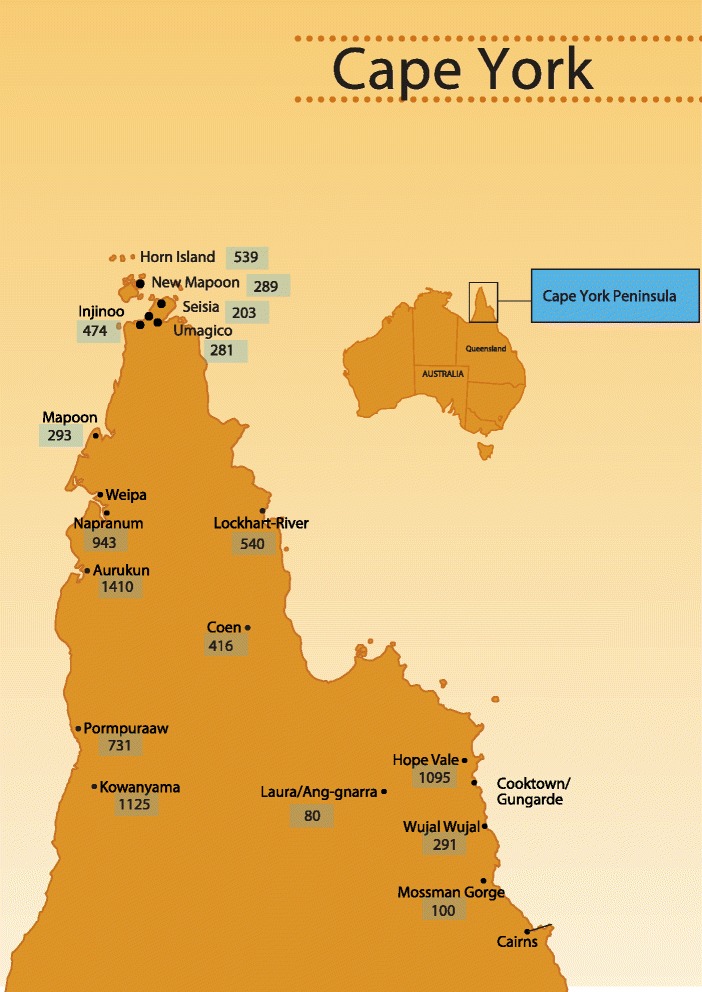
Map of Cape York Indigenous communities. Legend: Populations of Cape York communities.

The Baby Basket program was developed and implemented by Apunipima Cape York Health Council (Apunipima), the community controlled health organisation which provides primary health care services to the eleven Indigenous communities (Aurukun, Coen, Hopevale, Kowanyama, Laura, Mapoon, Mossman Gorge, Napranum, Lockhart River, Pormpuraaw and Wujal Wujal) (Figure 1). Apunipima has worked in partnership with Queensland Health and the Royal Flying Doctor Service to deliver antenatal care services, but is currently assuming control through a phased transition of responsibility for service delivery. Cape York women have a planned departure to Cairns Hospital (in the closest regional city) for birthing at 36 weeks gestation.

### Participants

Seven women who had received Baby Baskets and three family members were interviewed; and 18 healthcare workers participated in focus groups. The women were either pregnant or recently pregnant, were from six of the eleven Cape York communities and ranged in age from 21 years to 34 years (Table 1). Family members were aunts who accompanied the women to Cairns for birthing. Four women and all three family members were interviewed at Aboriginal hostels where they were staying since being transferred to Cairns in anticipation of the birth of their babies; two in their home community of Mossman Gorge; and one who had subsequently relocated to Cairns, at Apunipima.

**Table 1:** **Women interviewed, community of origin, age and Baby Baskets received**

**Participant**	**Community**	**Age**	**Where interviewed**	**BB1**	**BB2**	**BB3**
Expectant woman	Aurukun	late 20s	Hostel	y	y	y
	Mossman	34	At home	y	y	y
	Wujal Wujal	early 20s	Hostel	y	y	n
	Wujal Wujal	21	Mookai Rosie	y	y	n
	Hope Vale	mid 20s	Apunipima	y	y	y
	Mossman	23	Wellbeing Centre	y	y	y
	Lockhart River	24	Hostel	y	y	y
Family member	Kowanyama	40+	Hostel	n	n	n
	Kowanyama	40+	Hostel	n	n	n
	Cairns/Yarrabah	60s	Hostel	n	n	n

All but two of the 18 healthcare workers were employed by Apunipima; the others were employed by Mookai Rosie Bi-Bayan Aboriginal hostel which provides accommodation for women awaiting the births of their babies in Cairns. Healthcare workers from each of the 11 remote Cape York communities serviced by Apunipima were represented. Eight were Indigenous (Murri) health workers, a professional group who variously play clinical, liaison and cultural brokerage, health promotion, community care, administration and management, and policy development and/or program planning roles. There were also four clinical nurses, four midwives, one child family health nurse and one doctor. Eleven had provided Baby Baskets to Cape York women and sixteen had provided associated education.

### The intervention

The Baby Basket program aimed to facilitate engagement of women and their families with the health system. A total of three different baskets were delivered to each woman and contained items appropriate to her stage of pregnancy or early motherhood; in the first trimester, immediately prior to birth and in the first weeks post-birth (Table 2). The first and third baskets were provided in the women’s home communities whilst the second basket was provided in Cairns during their stay awaiting birth.

**Table 2:** **Contents of the Baby Baskets**

**Baby Basket #1**	**Baby Basket #2**	**Baby Basket #3**
**(Antenatal)**	**(Perinatal)**	**(Postnatal)**
Baby bed (sleeper)	Baby singlets (x5)	Sipper cup
Shampoo and conditioner	Breast pads	Plastic fork, spoon, plate
Fine tooth comb	Cotton baby wraps (x3)	Educational soft toy
Bunjalbi tailored health education book	Cotton wool balls	First aid kit
Soap and soap holder	Deodorant	Towel
Sorbelene cream	Emery board	Soap family pack (x6)
Toiletry bag	Baby grooming kit	Band aides
Tooth brush and holder	Hairbrush	Educational book
Toothpaste	Jumpsuits (x3)	Apunipima backpack
Washers	Maternity pads	
Apunipima tote bag	Moist wipes	
Five $40 fruit and vegetable vouchers	Cloth nappies (x6)	
	Nappy clips (x2)	
	Disposable nappies	
	Night dress	
	Shampoo and conditioner	
	Soap	
	Sorbolene cream	
	Tissues	
	Toothbrush and toothpaste	
	Towel	
	Washer	
	Baby soap	
	Zinc cream	
	Apunipima bag	

In conjunction with the material goods, the program also provided education about nutrition, exercise, smoking, alcohol-related behaviours and care for babies. Health education was provided primarily by Apunipima’s Indigenous health workers through a home visiting approach. But flexibly, it was also delivered by partner organisations (Queensland Health, the Royal Flying Doctor Service and/or Mookai Rosie Bi-Bayan), other professional groups (midwives or nurses) and in other places outside the home, particularly the clinic.

### Data and analysis

Consistent with grounded theory methods, theoretical sampling was used whereby data collection was directed by concepts arising in the evolving theory [14,16]. Two interview/focus group guides - for women and family members, and for healthcare workers - were developed based on the themes and principles from our prior literature review [8]. Questions pertained to women’s pregnancy and early child care; recall of the Baby Basket and associated education; understandings of the purpose of the program; useful or meaningful aspects; what was not useful; where (home or clinic) and when the program was received/delivered; whether family members were involved and/or education was shared with them; whether the program influenced visits to the clinic or not; and suggestions for improvement. Participants were encouraged to tell stories and provide examples of their experiences.

In keeping with other grounded theory studies involving Indigenous Australian participants, we anticipated sampling approximately 20 women and 10 healthcare workers [21,22]. Using the interview guide, three women were initially interviewed through face-to-face, in-depth semi-structured interviews lasting from 60 to 90 minutes from December 2013 to May 2014. A 90 minute focus group was also conducted with healthcare workers who were attending an in-service training workshop related to the Baby Basket program in Cairns. No identifiers or pseudonyms were applied and interviews and the focus group were recorded and transcribed verbatim. The data were coded in NVivo with the constant comparison method used to generate categories and progressively identify relationships between categories [16]. The interview guide was amended to probe further explanations of these categories and relationships at each additional interview. The process of examining the themes and their interrelationships was repeated until the theorist (JM) was satisfied that: 1) the higher order constructs and their relationships could be modelled in such a way that explained the great majority of the data, and 2) the common purpose and process underpinning Baby Basket implementation were identified [17]. After analysing the interview/focus group transcripts from seven women, three family members and 18 healthcare workers, we found that no new findings were emerging; hence theoretical saturation was reached [16].

## Results and discussion

**Figure 2:**
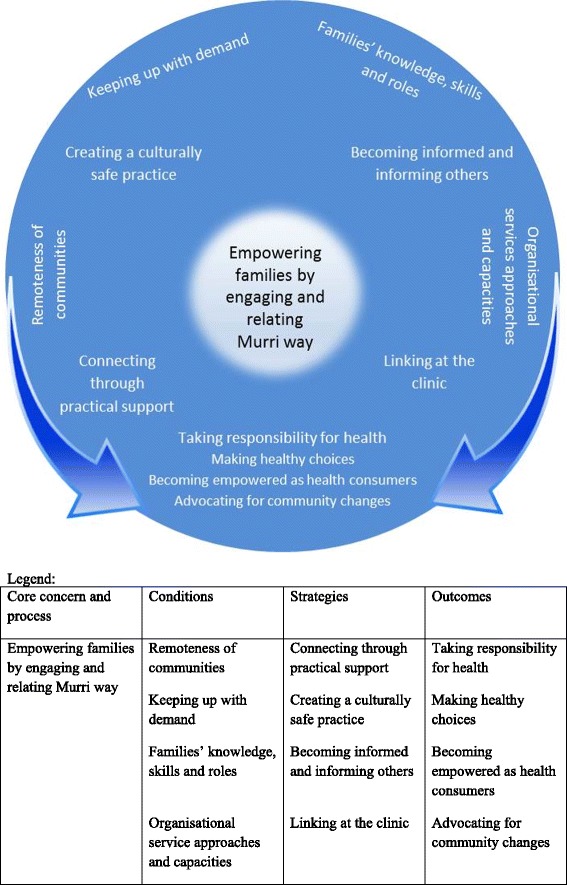
Theoretical model of *empowering families* by *engaging and relating Murri way*.

Depicted in Figure 2 are the interrelated common purpose and process by which Baby Basket implementation occurred (inner circle), social environment which influenced Baby Basket implementation (outer circle), strategies that combined to comprise implementation (intermediate ring) and consequences of program implementation (lower centre). The model and verifying grounded qualitative data are presented below. Theoretical terms are italicised.

Program implementation entailed *empowering families* through a process of *engaging and relating Murri way* between women, family members and healthcare workers. Key influencing conditions of the social environment were *the remoteness of communities*, *keeping up with demand*, *families’ knowledge, skills and roles* and *organisational service approaches and capacities. Engaging and relating Murri way* occurred through four strategies: *connecting through practical support, creating a culturally safe practice, becoming informed and informing others*, and *linking at the clinic*. These strategies resulted in women and families *taking responsibility for health* through *making healthy choices, becoming empowered health consumers* and *advocating for community changes*.

### Common purpose: *empowering families*

The common purpose for those who were active in delivering and receiving the Baby Basket program was *empowering families*. Empowerment was important for families to ensure that the women and their partners were confident and well prepared for the birth and parenthood so that babies had the best start in life. This was identified in the narrative of one woman who recalled: “I sat down with my partner and he was reading it (Bunjalbi Book included in the antenatal basket) and he’s like, ahhh. He’s a first time father as well you know”. A non-Aboriginal manager also reflected: “If you have a disempowered, sad woman, she is not going to be able to make any of those behavioural changes”. Empowering families was important not only because the Baby Basket aimed to better prepare women for their prospective roles as mothers, but also because extended family members were closely involved in caring for and supporting women and their new babies.

### Central process of implementation: *engaging and relating murri way*

The process of program implementation occurred through *engaging and relating Murri way*. The baskets provided an initial tool for engagement between women and their family members with Apunipima and other healthcare workers; and this set the foundation for relating Murri way. This referred to the local Murri (Indigenous Queenslanders) way of relating about health issues which involved “yarning” (an Aboriginal concept for coming together informally to share experiences and knowledges) in a holistic way about a woman’s or family’s wellbeing.

A healthcare worker spoke about the baskets as a “tool for engagement”; while another said “it’s almost like your ticket in”. Similarly, the women spoke of their engagement with healthcare workers as: “local faces at the clinic”; and “she [nurse] gave me the basket… she told me about the basket, she introduced me to the girls who were working for Apunipima”. Such perceptions of engagement contrasted with women’s narrations of the non-engagement experienced when they arrived at the regional hospital. For example, one woman said: “that cranky old lady that … don’t even talk to you; you just feel very uncomfortable”. The women appreciated the baskets’ contents and this appreciation established the basis for building relationships with healthcare workers.

Relating Murri way was critical to facilitating the provision of health care. One woman described her appreciation of the basket and the importance of relationships with healthcare workers in preparing women for birth and motherhood:

“the Baby Basket is beautiful to have, especially for women like us Cape York girls, like Indigenous women…its good because more of young girls are getting pregnant …. It’s sort of like a push; a sort of kick on the butt. It’s a good start for them you know.”

In the absence of relating Murri way, the women were sometimes reluctant to seek healthcare advice or assistance. An Indigenous (Murri) health worker reflected that many women would not disclose vital health information to healthcare workers with whom they had no relationship. For example, she recalled:

“On many occasions I’ve taken ladies with mastitis and urine infections. And they would have seen the EMS [Emergency Medical Services] lady that day. But I get the phone call and I take them… so it’s not that the EMS haven’t done their job. They (the women) just haven’t told them”.

An Indigenous health worker also spoke of the importance of relating Murri way with families, suggesting:

“It doesn't really matter who is in the household… because the older siblings will be looking after the kid, or the grannie, or the granddad. So when you do the education, regardless if mum not there, if she is at work or at the shops or something, it’s whoever’s looking after the kid as well who gets that education”.

For healthcare workers, relating Murri way involved a lengthy process of establishing trust and rapport with the women and their family members. For example, an Indigenous health worker said: “For me to relate to the people, I have to get to know them first. If I just chuck myself at them, they won’t bite it”. Thus, the engagement facilitated by the Baby Baskets led to the development and/or strengthening of relationships which provided a foundation for sustained health care through the processes of pregnancy, birth and early parenting.

*Engaging and relating Murri way* also occurred to some extent between the different professional groups within Apunipima’s transdisciplinary health team (doctors, nurses, midwives and Indigenous health workers); and between Apunipima and partner organisations. A non-Indigenous health manager spoke about the benefits of inter-cultural relating between Indigenous and non-Indigenous health professionals:

“The [Indigenous] health worker is learning at the same time, and the nurse is also learning what the best way is to approach a family and what the wording has to be, what the languaging is around things, what the traditional words are for Indigenous language and are appropriate for use in certain circumstances. So you know there is a lot that can happen in that fairly simple interaction”.

Such efforts towards culturally appropriate engaging and relating allowed healthcare workers to offer more comprehensive care by complementing each other’s skills and experience.

For women, *engaging and relating Murri way* also occurred with their partners, family and community members, and other women. For example, one pregnant woman provided support to another, recalling of the other woman: “she was getting scared…. And so I go ‘look we go in here, they have got the Apunipima staff here too. You sit down with them and they tell you stories about how big it is, and it’s not scary’” Through *engaging and relating Murri way* with each other, pregnant women assisted each other with taking on information about pregnancy, accessing healthy foods, linking with the clinic and other health enhancing behaviours.

### Conditions of the social environment

Although every Cape York community is different, four key aspects of the social environment affected program implementation. First was the *remoteness of communities.* Remoteness influenced program provision because of the lack of routinely available pregnancy and baby goods in remote communities. Remoteness also affected families’ ability to make healthy choices because many remote communities lack supplies of affordable fruit and vegetables; what is available is often of poor quality and not affordable. One woman recipient of a Baby Basket commented: “…they will have problems down the track like diabetes, and all these other disease coming along in their pregnancy due to lack of supplying fresh fruit and vegies.” Remoteness also required the universal planned departures of pregnant women from communities at 36 weeks pregnancy to give birth in Cairns. Departures resulted in their separation from family and community support at this critical time, and necessitated additional support from health services.

The second aspect affecting program implementation was *keeping up with demand* due to the high birth rate, prevalence of risky pregnancies and preventable childhood diseases within these communities. One woman recalled:

“Me and my friend didn’t know we were pregnant, well this girl up here is 3 weeks in front of me. All of a sudden, we went to the clinic. One found out she was 6 weeks - another 2, and 3 weeks later I found out I was 8 weeks pregnant…. next minute, you see all these little tribe running around”.

The high birth rate in remote communities created a strong demand for Baby Basket; particularly in large communities where healthcare workers were stretched to keep up. A health worker narrated her experience of delivering the program in a large Cape York community:

“Did I do the Baby Basket then, oh no, did I do this other one? Shit. There’s other kids - did we get them for their immunisations and child health checks? You know what I mean? It’s mind boggling the amount of women that are pregnant and the amount of kids that are tiny”.

Further, the health issues mentioned by women and healthcare workers included high blood pressure, an ear infection, multiple miscarriages, rheumatic heart disease and gestational diabetes during pregnancies; as well as haemorrhages, and mastitis post-birth. Hence, while the approach of Apunipima was to focus primarily on the women’s and baby’s wellbeing and the family as a whole, many women had risky pregnancies that required medical intervention.

High rates of chronic and infectious diseases also posed challenges for healthcare provision. The common health issues affecting young children were described by an Indigenous health worker:

“…ear health, skin, so scabies, impetigo, are mainly my ones. I don’t know about any other communities but we do, I mean we gave four bicillins in one day to children, like it was that bad. Head lice, eye…”.

The prevalence of preventable diseases reinforced the need for the Baby Basket program; particularly the health education to women and their families.

Third was *families’ knowledge, skills and roles* in the community, and specifically extant family and/or work responsibilities. For example, one woman reflected:

“I got my own place; I look after my little brother and sister too … My mum passed away when my little one was 9 months…. No one else is going to look after them, so it’s like, I don’t want to see my little brother and sister going to people that won’t help them.”

Such relationships and responsibilities influenced women’s and families’ needs, capacity and choices with regard to their engagement with the program.

Fourth were *organisational and service approaches and capacities.* The family-centred and community–controlled approach of Apunipima was appreciated by the women. One said:

“It’s really sad when culture dies. Our main person is sick now, old fella, Elder is sick…. that’s why we are trying to get to our youngsters. That’s why it’s really, really good that our partnership with Apunipima and other you know… is Indigenous based”.

Healthcare workers also recognised the influence of Apunipima’s organisational values and processes on work practices. One nurse acknowledged:

“I’m sitting feeling very new to this organisation [Apunipima] having worked somewhere else. The way the whole Baby Basket concept was explained is completely different to everyone’s understanding here. I mean, it’s just different … you’re working in a different capacity…”

Delivery through the “different” community-controlled health-worker-led, family-oriented and home visiting approach enabled clinic staff to build good rapport with women and family members and facilitated implementation that was responsive to community needs and feedback.

### Strategies

*Engaging and relating Murri way* occurred through four strategies which are presented sequentially, but in practice they overlapped. They were: *connecting through practical support, creating a culturally safe practice, becoming informed and informing others*, and *linking at the clinic*.

#### Connecting through practical support

*Connecting through practical support* refers to the initial engagement between women and healthcare workers facilitated by the provision of the pregnancy, birth and baby goods. Women and healthcare workers considered the items provided through the Baby Basket program to be useful, and appreciated their contribution to preparing women for their changing roles and responsibilities in their families. One woman said:

“…they come in handy. The first one is really good, they provide for mothers, all this stuff you need … the second one … they meant to give us before we go in, it’s got all the baby singlet, clothes, basket, shampoo, singlet, their own towel. I felt over the moon. I thought that’s really good”.

Women suggested that the Baby Baskets were particularly useful for supporting those who were likely to be less well prepared for birth and motherhood – for example, those who were having their first baby, were young and/or required emergency evacuations from communities. Another woman commented:

“… their first baby, they don’t have anyone to support them, you know, it’s really good. It comes in handy…It’s sort of like a showing of the idea of what baby needs, and for themselves, before baby born and after he or she born, you know.”

Although the composition of baskets had been relatively stable since 2009, the women recipients were aware of the small amendments over time. Women also suggested further improvements to the contents of the baskets; primarily additional or amended material items and educational resources. The awareness of amendments and consideration given to recommended changes suggested the high value placed by women on the baskets.

#### Creating a culturally safe practice

Indigenous health workers engaged with the women and families by *creating a culturally safe practice.* They did so by *engaging and relating Murri way* responsively and in a respectful and inclusive manner displaying patience, flexibility, confidentiality and communication skills. Indigenous health workers generally work in their community of origin; hence they have an intimate knowledge of the local social and cultural dynamics. An Indigenous health worker described the flexibility required in responsively relating with women in ways that were culturally safe. She commented: “They’re young and you just having to listen to what people complain about. It may not even be relevant to your job but you’re there, you’re listening.” Another health worker explained that language was critical to families’ interpretation and understanding of health messages: “Yeah we usually speak more Kriol to them and they understand it much better”. The importance of language was also illustrated by use of plain English and use of appropriate visual images in the educational resources (such as the Bunjalbi Book) provided to families.

Non-Indigenous healthcare workers also recognised the critical importance of Indigenous health workers in creating and maintaining that space and ensuring that the women understood the health education and care provided. A midwife talked about the ways she worked with an Indigenous health worker to better understand the women’s needs and ensure better care:

“Often when we sit there and explain things, the ladies will sit there and go ‘yes, yes, yes.’ it’s only when [names of two Indigenous health workers] have gone out after me and they’ll come back and say ‘actually when you said that … they didn’t quite get that’.…You know, and that’s where we learn so much”.

Home visiting was a key strategy for *creating a culturally safe practice*. This strategy was endorsed by both healthcare workers and women so long as it accorded with women’s preferences. The women experienced home visiting as useful for relieving the stress of having to obtain transport to get to the clinic. An Indigenous health worker considered that home visiting allowed more personal care and enhanced trust: “to me it is like you care, from being in an Aboriginal community, it’s like you come out of your comfort zone and you actually are in their environment and you see what they are going through”. A midwife noted: “the quality of the information you get about a family and how that family operates is just like the difference between a couple of words of description and a picture. It’s hugely different.” However, the logistics of providing three baskets posed challenges in relation to: “too much stuff to carry” outside the clinic; and many healthcare workers commented on the need for access to a dedicated vehicle. The home visiting approach needed to be administered flexibly to account for the varied preferences of women and families and varied cultural customs, and required resourcing.

*Creating a culturally safe practice* meant that care extended to women’s and families’ social and emotional wellbeing. An Indigenous health worker observed: “They’re too shamed to talk about their social emotional sides, whereas with us they are so connected to us, because they know us”. *Engaging and relating Murri way* through *creating a culturally safe practice* enabled discussions about sensitive issues such as cigarette smoking, marijuana use, alcohol and other health and wellbeing issues. For example, in relation to foetal alcohol syndrome (FAS), one Indigenous health worker said that she:

“…felt a bit uncomfortable with talking about the FAS, and they didn’t know anything about it. And some of them go really quiet because they may have drunk early. And I feel like it might be too late to be talking about that. But it is good. Then they’ll know for next time.”

Being assured that confidentiality would be maintained was critical. An Indigenous health worker commented: “I always tell them I can lose my job if start talking to youse about family after hours. So I reinsure that everything is kept confidential”. Thus, the knowledge, qualities and skills of Indigenous health workers, and flexible home visiting approaches allowed healthcare workers to *create a culturally safe practice*, thereby enabling discussions about sensitive, but necessary, discussions about issues that affected the health and wellbeing of the women and their babies.

#### Becoming informed and informing others

Within the space nurtured by employing culturally safe practice, women, partners and their families *became informed and informed others* about prevention and care; this was the third sub-process of *engaging and relating Murri way*. Women, partners and their families learnt what to expect through the processes of pregnancy, birth and motherhood, how to prevent ill-health, care for themselves and their children, and passed on their learnings to other pregnant women. A health manager commented on the value of engaging partners and other family members:

“…five pairs of ears listening rather than one; including the one who does the cooking and the one who decides who is going to sleep where. And all those things that are really important regarding early life experiences.”

Several women said that they did not know much about pregnancy, birth or child rearing before becoming pregnant. One said: “I wish I’d had done this before. When my first baby. At least I would have known.” Another said: “my mum never talked to me about stuff like this. You know even teenager or growing up”. And yet another woman recalled:

“when we found out we were pregnant, we got our Baby Baskets, and the midwife and you know, Apunipima will tell us um, about the fruit and vegie…. and knowing the safety of the baby, and yourself, and not hurting yourself.”

Once informed, women were keen to share their knowledge to support other family and community members. An Indigenous health worker observed: “they’ll tell others… they’ll pass the word around”.

All healthcare workers said they provided education about safe sleeping for mum and baby; healthy food and nutrition; breastfeeding; general hygiene; smoking, alcohol and other drugs during pregnancy; and wellbeing. However, the entrenched nature of some health issues meant that even though education was regularly provided, healthcare workers remained concerned about its effectiveness in shifting behaviours. For example, a midwife said: “certainly we are giving out those messages about scabies and hearing health and all the rest of it every time… [but]… it’s long term and these issues have been around for a long time.” She continued: “And whether they take it on board is totally different”. Further, they expressed concern that the health promotion messages provided were at the discretion of individual healthcare workers and there was no consistency across the program. Hence, for example, a midwife commented:

“If you’re someone who’s a newish nurse to child health and you’ve never had children yourself and you’re sitting there thinking: ‘Oh God, I’m really not too sure what a six month old eats, but I’m hoping I’m giving the right advice… We can’t be sure; especially if … there is nothing to say that it is okay.”

In order to provide effective prevention and care messages, Indigenous health workers, nurses and midwives practiced two-way learning across roles. A health manager reflected: “the health worker is learning at the same time, and the nurse is also learning what the best way is to approach a family”. However, the concern about whether consistent health messages were provided suggested the need for a revised approach including training to inform healthcare workers about the focus and approach to *becoming informed and informing others*.

#### Linking at the clinic

The fourth sub-process, termed *linking at the clinic*, referred to strategies to encourage women, their partners and children to access the primary healthcare clinic for health screening, checks and treatment and care through continuing the process of *engaging and relating Murri way*. One of the core aims of the Baby Basket program was to encourage women to attend the clinic earlier for antenatal care. But with young or first mothers in particular, there were barriers to clinic attendance which required careful management through Indigenous health workers’ home visits and community knowledge. An Indigenous health worker reflected:

“We don’t normally catch them very early. Like we’ve got two ladies that are young; like one is probably 17 or two of them are 17, and their bellies are out. So we hear from family that they are pregnant but they never come to the clinic yet. So I don’t know how long they want to hide it for”.

One healthcare worker mentioned that some women were happy to visit the clinic because of the rapport staff built with the women and family members. The employment of Indigenous staff at the clinic was important, with one Indigenous health worker noting: “Some women are in shame for baby, and they go to clinic for check; black nurse they’re alright”. This quote refers to the shame or embarrassment felt by insecure young or new mothers resulting from internalisation of the historical disempowerment of Indigenous Australians; and their perception that other Aboriginal healthcare workers would understand their parenting concerns. However, clinics were not always an appropriate environment in which to provide Baby Baskets. A midwife said:

“you’ve done all this stuff and it’s like ‘oh baby basket’. It’s like an afterthought… Because it was being done in a very busy clinic, where you were very aware who was waiting outside and all the other demands that were upon you”.

Thus, the role of clinics was critical for provision of care, but not a preferred option for handing out the baskets or associated education.

Postnatal visits were important for checking for and treating the diseases experienced by Cape York children and caring for women’s postnatal health. For example, an Indigenous health worker spoke of: “…a boy who is probably eight months, not even that. He was rid with scabies”. Another described an after-hours conversation with a client:

“I had a lady pull me up in the shopping centre and she said ‘Oh, sis, bubby’s not attaching properly, like it’s sore, it hurts when he gets on my susu… I’ve been sick and tired and sleeping all day’ and I said: ‘now darling you really need to go to the hospital. Like now! …It sounds like you got mastitis…’.”

This conversation exemplifies the importance of multiple strategies to enable *linking at the clinic* through the foundational relationships between healthcare workers and women in facilitating healthcare provision.

### Consequences: taking responsibility for health

Baby Basket implementation supported women, family members and healthcare workers in *taking responsibility for health*. While acknowledging that it was the women’s right to take education messages on board and to change behaviour, health care workers were heartened by examples of women and families *making healthy choices*. One woman talked about how pregnancy prompted her choice to stop smoking:

“At first I was a really heavy smoker. Like two packets a day… Yeah and when I found out I was probably only three weeks pregnant. And so I gave up straight away. I gave up for two years before I started back up again…Yeah I was really surprised myself”.

Another woman reflected: “I ate heaps of vegies! I would only eat it if it was steamed”. Such examples of behaviour change were heartening.

Women also experienced *becoming empowered as health consumers,* taking control of their pregnancy, birthing and mothering journeys with the support of the Baby Basket program: “Just learning what happens when your baby comes, or what to expect in labour.” Another woman recalled: “I didn’t want to go through with the pregnancy there…the hospital wasn’t very clean, so I decided to come back home.” Being prepared for the births of their babies’ relieved stress and shame for women, and provided a better start for newborns.

*Taking responsibility for health* was also observed in women *advocating for community changes* to improve the wellbeing of other women. One woman worked as a youth worker, and mentioned the important role of providing contraceptive advice and condoms to young people who wished to prevent pregnancy. Two women commented about the availability of quality and affordability of fruit and vegetables in the stores in remote communities; one saying:

“Whenever I go in the shop, I fight for the health of young women, pregnant women, you know, women that are old. I say, ‘why do you put out rotten fruit on the shelf… We get young women who are having babies, and they need fresh fruit and vegies’”.

Two women mentioned that they wished that the baskets could be more widely available so that others could also enjoy their contents. One said: “every mum should be able to get it”. This appreciation by recipients, as well as the willingness and capacity of health providers to deliver Baby Baskets suggests the feasibility of transferring the approach to other sites.

### Limitations

A limitation of this study arose from the challenges faced in accessing women who had received the Baby Baskets and who were willing to be interviewed. The women interviewed in Cairns were at least 36 weeks pregnant, were displaced from their normal family and home support structures, and were likely to have had other priorities in preparing for the forthcoming births of their babies. Hence, repeated attempts and arrangements to interview some women were fruitless. Nevertheless, one of the strengths with grounded theory methods is that it allows for the concurrent collection, analysis and development of theoretical codes and categories – this prevents the collection of unnecessary data once saturation is reached.

## Discussion

Family-empowerment approaches have been advocated for the last decade as options for improving the primary prevention of Indigenous chronic disease [23] and mental health [24,25]. In the mainstream literature, family empowerment has also been touted as critical in relation to chronic disease as well as mental health, disability and juvenile offending [26]. However, despite the intuitive appeal of family-empowerment approaches in Indigenous maternal and child health, these have not previously been theorised or evaluated.

Drawing from Wallerstein [27] we conceptualise family empowerment as a process by which families gain mastery or control over their lives, and particularly the self-regulation of health management and negotiation of the health and other systems to meet the needs of the family. Explicit family empowerment processes encompass changed attitudes and skills for reducing stress, dealing better with the challenges of daily life, the adoption of healthier lifestyles and more active participation in society [24]. Family-centred approaches make sense since the family is the constant in a child’s life, while particularly in remote Indigenous communities, health service staff and systems are transient. Additionally, fathers parenting behaviors and styles can be as much, if not more, associated with early childhood health risks (such as preschooler overweight and obesity) as mothers’ parenting behaviours [28].

The theoretical model of family empowerment developed through this research project provided guidance to Apunipima for quality improvement to amend the program design of the Baby Basket program and develop a revised “Baby One Program”, in its stead. The provision of baby baskets was incorporated within the new Baby One Program, but recognition of the importance of *empowering families* as part of the improvement of maternal and child health outcomes led to an extension of Apunipima’s home visiting approach and provision of consistent care for women, babies, partners and the wider family from pregnancy through to birth and up to 1000 days (the child’s third birthday). The theoretical conceptualisation of *engaging and relating Murri way* as the central process by which program implementation occurred, and recognition of the lead role of Indigenous health workers led Apunipima to formally recognise and train Indigenous health workers as Baby One Program leaders. The in-service training program and a comprehensive Baby One manual provide resources for working towards greater consistency in the provision of education messages across the Cape. The Indigenous health workers now use a case-load model to work with parents, children and families in collaboration with midwives, child health nurses, allied health practitioners and the health manager. This conceptualisation of the central role of Indigenous health workers in maternal and child health has also not previously been theorised in the literature. Indigenous Australian and international research studies have found that midwife-run patient centred antenatal programs result in greater patient satisfaction, better use of antenatal services and improved health outcomes for mother and baby; but that GP models of antenatal are not as effective [29]. There have not been conclusive studies about the effectiveness of Indigenous health worker-led maternal and child health programs; the effectiveness of Indigenous health workers in leading program implementation of the Baby One Program will need to be further tested.

## Conclusions

In summary, within the remote Cape York communities, the Baby Basket program was implemented for the purpose of *empowering families* through the process of *engaging and relating Murri way* between women, their families and health service providers. The theoretical model was used for guiding program improvements and to consider the potential for transferring the approach to other sites. Program acceptability and appreciation in Cape York suggested that with adequate resourcing, it was likely to be transferable to other regions of Australia with similar social environments and sustainable in those sites. However, program implementation would need to be based on *empowering families* by *engaging and relating* with Indigenous pregnant women and their families in the local Indigenous style. The theoretical model of Baby Basket implementation is also likely to be applicable to other global Indigenous maternal and child health programs that aim to promote improved engagement with primary health care services.

## Endnote

^a^Data was available for only New South Wales, Queensland, South Australia and the Northern Territory.

## Abbreviations

Apunipima: Apunipima Cape York Health Council
NVivo: Qualitative data analysis software
